# Global health and social accountability: An essential synergy for the 21^st^ century medical school

**DOI:** 10.7189/jogh.11.03045

**Published:** 2021-03-01

**Authors:** Michael Fitzgerald, Esther Shoemaker, David Ponka, Mark Walker, Claire Kendall

**Affiliations:** 1 C.T. Lamont Primary Health Care Research Centre, Bruyère Research Institute, Ottawa, Canada; 2 ICES, Toronto, Canada; 3 Ottawa Hospital Research Institute, Ottawa, Canada; 4 Institute for Global Public Health, University of Manitoba, Winnipeg, Canada; 5 Besrour Centre for Global Family Medicine, College of Family Physicians of Canada, Toronto, Canada; 6 Department of Family Medicine, University of Ottawa, Ottawa, Canada; 7 Department of Obstetrics, Gynecology and Newborn Care, The Ottawa Hospital, Ottawa, Canada; 8 Department of Obstetrics and Gynecology, University of Ottawa, Ottawa, Canada; 9 Li Ka Shing Knowledge Institute, St. Michael’s Hospital, Toronto, Canada

Global health and social accountability are two endeavours of 21^st^ century medical schools that set them apart from their Flexnerian predecessors. In the Flexnerian model, medicine was approached as a scientific discipline, and thus the curriculum emphasized basic science and laboratory methods [1,2]. The new endeavours signal a shift in medical schools’ approach to health to include a focus on biopsychosocial factors [1], social determinants of health, and health equity. In this commentary, we argue that when global health and social accountability each draw on the strength of the other, these endeavours can be reinforced in a powerful synergy that enables them to realize their full potential.

## SOCIAL ACCOUNTABILITY

The social accountability of medical schools has its provenance with the WHO, which defined it in 1995 as “the obligation [of medical schools] to direct their education, research and service activities towards addressing the priority health concerns of the community, region, and/or nation they have a mandate to serve. The priority health concerns are to be identified jointly by governments, health care organizations, health professionals and the public” [3]. Over the past twenty-five years, Boelen and his colleagues have elaborated on the significance of this definition. Particularly interesting is the notion of social obligation, with its differentiation of social *responsibility* (simple awareness of and interest in health inequities and social determinants of health), social *responsiveness* (engagement in activities to address health inequities and social determinants), and social *accountability*, the latter being higher on the gradient and so incorporating the other two. Social accountability differs from the other positions in that it involves working collaboratively with stakeholders and especially the public, civil society, or communities [4]. What interests us is the part of the definition that often gets overlooked, ie, engagement with communities [5], which suggests that social accountability is really about being accountable to the people that a medical school is aiming to help by addressing their health needs. And being accountable requires, in the first instance, engaging with people to find out what their health needs and their health priorities are.

**Figure 1:**
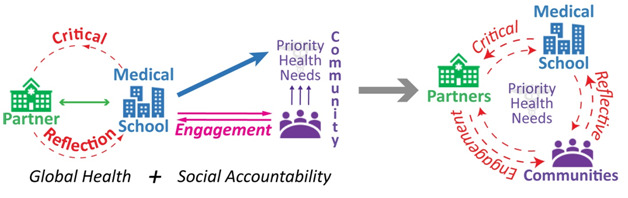
Global health and social accountability synergize for critical reflective engagement.

The challenge for the 21^st^ century, post-Flexnerian medical school is how best to engage with communities in a way that ensures that that they themselves can articulate their own health priorities. Unless medical schools do engage in this way, they run the risk of presupposing or predetermining those needs quite unaccountably. Arguably, medical schools haven’t always been good at engagement, partly because in the 20th century they tended to follow the Flexnerian model, with its strong biomedical focus. As we see it, part of the problem is that there is at times a lack of sufficient reflection on the position of the medical school in society and its power and influence, and how these structural elements affect engagement. We suggest that medical schools could use more critical reflection in their social accountability initiatives, which is where we see global health, another element essential to the post-Flexnerian medical school, enhancing social accountability (**Figure 1**).

## GLOBAL HEALTH

Global health education in medical schools typically has two main parts: (1) a theoretical, critical, and reflective component found, for example, in academic courses, that encompasses learning opportunities on the history and practice of global health; and (2) a practical element that encompasses placements or global health electives both in local (usually underserved) communities, and abroad in low-/middle-income countries. Looking at the theoretical component, we find extensive critical reflection on the origins and meaning of global health, and its roots in colonialism, tropical medicine, developmentalism, and globalization. A variety of theoretical and conceptual standpoints are employed in these analyses, in which issues of power, position, and agency feature prominently.

We propose that medical schools would greatly enhance their social accountability (or improve their position on the social obligation gradient) if they were to listen closely to this critical global health approach and incorporate its lessons into their engagement activities. This could help medical schools achieve more authentic and enduring engagement with their communities and other stakeholders.

Although, theoretically, global health education has a strong focus on social determinants of health and health equity [6], the practical component is rather more biomedically oriented, for example, in focussing on issues such as diseases and conditions unlikely to be encountered at home (ie, in resource-rich countries [7]). The elective experience also tends to prioritize the medical trainee’s own experience [6-8]. Thus, what is noticeable with this component of global health in medical schools is that often it is not all that oriented towards the needs and priorities of the communities where they take place – or at least, not as much as it could be. We think that this is partly because global health electives don’t have community engagement built in as a fundamental component, and that with a greater focus on engagement, global health electives would more effectively teach the biopsychosocial side of health, and also help medical schools fulfill their social accountability mandates. We recognize that, at least in North America, many educational programs in global health have their homes outside of medical schools, in arts or health sciences faculties. However, our focus here is on the transformative potential of this component for medical education in relation to the concept of social accountability, which as formulated by the WHO in 1995, refers explicitly to medical schools [3]. And, as Watterson et al. point out, the vast majority of medical schools around the world do include at least some global health elements in their curricula [6]. Global health, then, can learn from social accountability as well. Combining the strengths of these two domains and their respective approaches to health and embedding them in the mission and role of the 21^st^ century medical school would facilitate the development of *critical reflective engagement*.

**Figure:**
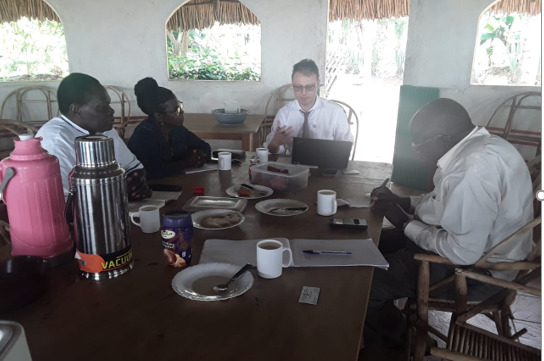
Photo: Community consultation on mental health integration into primary care, Asembo Bay, Kenya. Courtesy of the Besrour Centre for Global Family Medicine (used with permission).

## EXAMPLES

One example that illustrates a critically reflective approach to community and stakeholder engagement is the University of New Mexico Health Sciences Center’s (UNMHSC) development and implementation of Health Extension Rural Offices (HEROs) and its “Vision 2020” concept [9,10]. This was a response to the poor health status of the New Mexico population, and the discovery (from rural community health leaders) that the institution was regarded as inaccessible and having a limited interest in communities. As a result, the UNMHSC developed a network of HEROs located in communities, who could act as facilitators “linking community health priorities with UNMHSC resources” [10]. The HEROs enabled a more effective way of addressing social determinants of health in the different communities, but also led the institution to realize the mismatch between community health priorities (predominantly biosocial) and institutional research interests (predominantly biomedical). One result of the introduction of HEROs has been “a notable attitudinal shift at UNMHSC regarding forming alliances with community partners, sustaining presence in and relationships with communities, and incorporating social determinants as legitimate areas for investigation, education, and clinical service” [10].

An example that illustrates a community engagement approach to global health electives is McGill University’s Chilcapamba to Montreal Global Health Elective (CMGHE), which takes place in Ecuador [11]. This program, which began in 2008, uses participatory research for community engagement, and incorporates research into its eight-week placements. Community-identified priorities are the basis for trainee recruitment, community leaders supervise trainees during their placements, and at the end of the project, trainees produce a lay summary of the preliminary results for community dissemination. Overall, the program has resulted in increased community capacity through the attention paid to power differentials, to ensuring community leadership, and to a bidirectional flow of knowledge. One specific outcome has been the development of a Community Health Worker Training Program in the host communities.

## CONCLUSION

As we have argued in this commentary, and as the examples above show, a critically reflective approach to combining social accountability and global health can make possible true interaction and partnership with communities and other institutions to support the practical components of community health in ways that truly address global health inequities.
